# Effect of Cerium Content on Non–Metallic Inclusions and Solidification Microstructure in 55SiCr Spring Steel

**DOI:** 10.3390/ma17225450

**Published:** 2024-11-08

**Authors:** Haiyan Tang, Sen Cai, Peng Lan, Yu Ma, Yuhang Wang, Kaimin Wang

**Affiliations:** 1 School of Metallurgical and Ecological Engineering, University of Science and Technology Beijing, Beijing 100083, China; tanghaiyan@metall.ustb.edu.cn (H.T.);; 2 Capital Engineering & Research Incorporation Limited, Beijing 100083, China

**Keywords:** rare earth, spring steel, inclusions, solidification structure, microstructure refinement

## Abstract

The effect of cerium content (0, 0.011, 0.017, 0.075 wt%) on non-metallic inclusions and solidification microstructures of 55SiCr high-strength spring steel was experimentally studied, along with thermodynamic calculations. The results show that Ce addition changes the type and size of inclusions in this steel and influences the characteristics of the solidification microstructure. In the sample without Ce addition, the main inclusions are MnS, SiO_2_, SiO_2_–MnS, and CaO–SiO_2_–MgO, and the equiaxed ratio in the solidification structure is 44.63%. However, when Ce content increases to 0.011 wt%, the inclusions in the steel become mainly Ce–S, Ce–O–S, and a small amount of MnS, and the equiaxed ratio increases to 50.42%. As the Ce content increases to 0.017 wt%, the inclusions are predominantly Ce–S, Ce–O–S, and Ce–O–S–Ca, while some Ce–P and Ce–O–P–C inclusions are also observed. The equiaxed ratio increases to 67.63%, showing the best effect on heterogeneous nucleation during solidification. When Ce content in the steel reaches 0.075 wt%, the Ce-containing inclusions are Ce–S, Ce–O, Ce–P, Ce–P–O, and Ce–O–S–As, and the size becomes larger. The formation mechanism of inclusions is explained by Gibbs free energy calculations and thermodynamic diagrams.

## 1. Introduction

Suspension springs and engine valve springs in automobiles are the most typical high-end applications for 55SiCr spring steel. Valve springs are core components of automotive engines, and their performance determines the final stability and reliability during service. Due to the severe working environment, the requirements for the fatigue performance of spring steel are extremely harsh, with the fatigue life needing to exceed 23 million cycles, equivalent to infinity [1]. A large number of studies [2,3,4] have shown that large-sized inclusions and matrix microstructures are the main reasons contributing to spring fatigue failure. Rare earth (RE) elements have the potential to modify the inclusions in steel and adjust the solidification microstructure and thus are increasingly used in metallurgical processes these years, becoming a hot research topic.

Wang et al. [5] added rare earth Ce to IF steel and found that the cluster-shaped Al_2_O_3_ inclusions were transformed into spherical or spindle-shaped rare earth inclusions with sizes of 2–5 μm. Geng et al. [6] studied the modification effect of Ce on the inclusions in a deoxidized steel during the Ruhrstahl–Heraeus (RH) refining process and showed that liquid Ca–Al–O inclusions can be changed into Ca–Ce–O–S + Ca–Al–Ce–O complex inclusions. Liu et al. [7] studied the effect of Ce–La complex alloy on inclusions in a 110-grade oil casing steel by Al-killed and Ca treatment process. The results showed that the inclusions in steel without rare earth addition are mainly Ca–Al(–Mg)–O, Ca–Al(–Mg)–O+CaS, CaS, and TiN. However, after adding the Ce–La alloy, they become mainly Ce–La–O(–S), Ce–La–O(–S) + CaS, Ce–La–P–As, and TiN.

For the inoculation function of RE alloying, Deng et al. [8] found that RE inclusions have a low degree of mismatch with ferrite, which can induce the formation of acicular ferrite within the grain and increase the volume fraction. Li et al. [9] showed that Ce can effectively increase the equiaxed ratio in the solidification structure of Fe–Mn–C–Al TWIP steel. When the superheat of molten steel is 50 °C and the Ce content is 0.064 wt%, the equiaxed ratio increases by 24% compared with the RE-free sample, and the average grain size decreases from 480 μm to 260 μm. Zhong et al. [10] added rare earth yttrium to 20SiMn steel and found that the thickness of the martensite lath and the spacing of the pearlite lamellae decreased with the increase in yttrium content, while the yield strength increased accordingly. Chu et al. [11] added 0.005% Ce to stainless steel, and the average grain size of the steel decreased, leading to an increase in tensile strength, yield strength, and impact toughness. Sun et al. [12] found that RE has a refining effect on the pearlite lamellae, as well as on the quenching and tempering microstructures of 65Si2CrV spring steel. The addition of 0.0039 wt% Ce can achieve the highest tensile strength and yield strength.

Numerous studies have confirmed that there is a reasonable range in the amount of REs [13,14,15,16,17,18] in steel, but the target amount varies in different steel grades and microstructure performance requirements. If rare earth is added in low amounts, its modification effect is not obvious, while a high amount will promote the generation of large-sized rare earth inclusions, deteriorating the performance of steel and resulting in nozzle clogging in industrial production. The application of RE in 60Si2Mn spring steel has been partially studied [19], but its application in the high-strength 55SiCr spring steel has rarely been reported. This study takes 55SiCr spring steel as the basic material, aiming to explore the influence of RE content on the inclusions and solidification structure in the matrix and to determine the reasonable amount of RE added to potentially increase its strength and fatigue life. It is expected that this study can provide a fundamental guideline for the industrial application of RE in 55SiCr steel.

## 2. Materials and Methods

**Figure 1:**
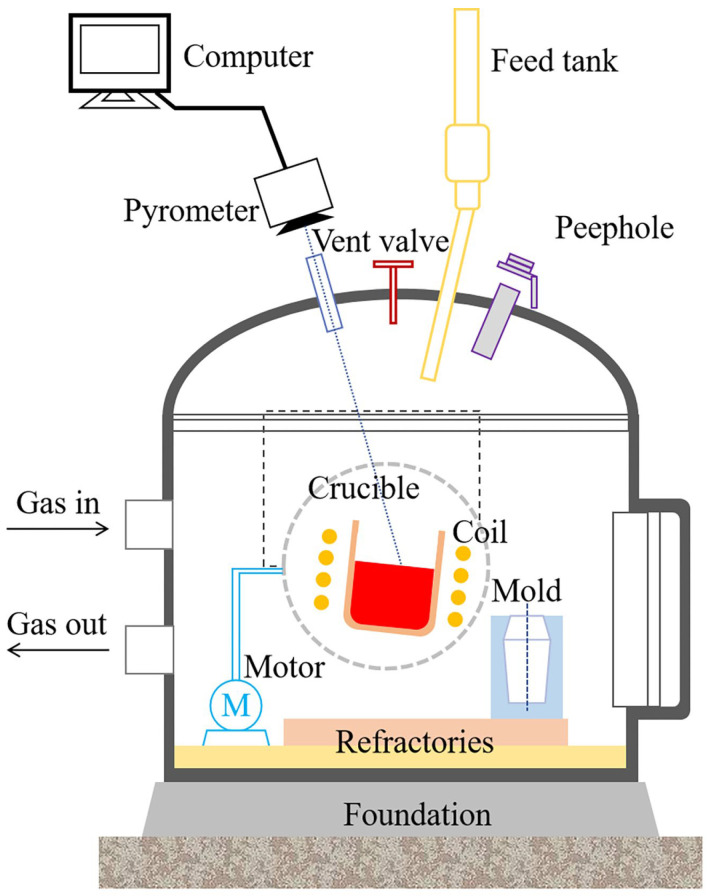
Schematic of experimental apparatus.

The basic material was the continuously cast billet of 55SiCr spring steel, which was produced by an industrial route of 150 t EAF–LF–VD–CC in a special steel plant. The Si–Mn alloy was employed for deoxygenation in the refining process. The Ce alloying experiment was carried out in the laboratory, and the experimental apparatus is illustrated in Figure 1. A 2 kg vacuum induction furnace (KZG-2) was adopted for steel melting, alloy addition, and sampling. Approximately 1300 g of weighed steel material was placed into a MgO crucible, which was then positioned inside the vacuum chamber. The furnace lid was closed to ensure that the entire system was well-sealed. The chamber was evacuated using a vacuum pump and subsequently backfilled with high-purity argon gas to reduce the oxygen pressure in the chamber and prevent reoxidation. The power switch was adjusted to increase the temperature at a rate of 2 kW every 10 min. Once the temperature reached 1600 °C, the target amount of Ce–Fe–Si alloy (25.8 wt% Ce, 57.5 wt% Fe, 16.7 wt% Si) was rapidly added to the molten steel through the feed tank and stirred. After a 10 s reaction period, the molten steel was poured into a pre-set mold and then air-cooled to room temperature. The Ce alloying scheme is shown in Table 1. The reason for selecting more than 0.01 wt% Ce content and 10 s holding time in molten steel is to simulate the mold-feeding process. Generally, there are three operation stations for the industrial addition of RE in the steelmaking process: refining ladle, tundish, and mold. The first two methods have a low RE yield and a risk of nozzle clogging during continuous casting, while feeding RE wire in the mold is optimal to simultaneously modify the non-metallic inclusions in steel and refine the solidification microstructure by RE.

**Figure 2:**
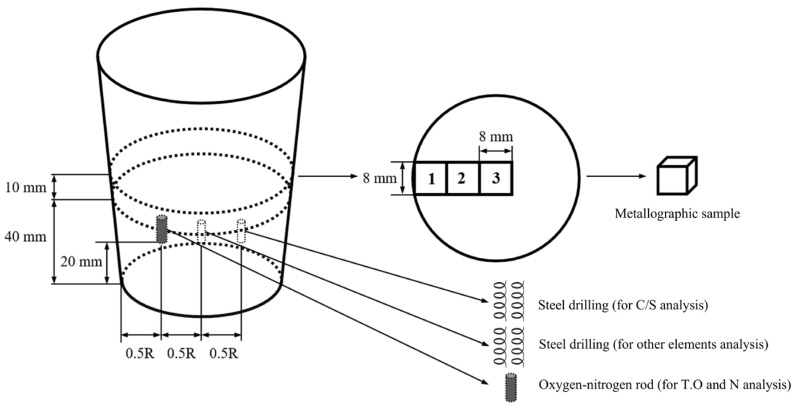
Schematic diagram of specimen processing.

**Figure 3:**
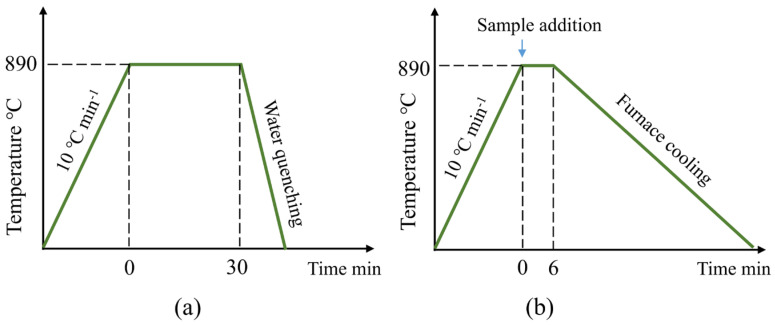
Schematics of two heat treatments for (**a**) No. 1 sample and (**b**) No. 2 sample.

The schematic diagram of specimen processing is illustrated in Figure 2. A cylinder rod sized φ5 mm × 7 mm was taken from a location about 20 mm from the ingot bottom for oxygen and nitrogen content analysis using the TCH600 oxygen & nitrogen analyzer (LECO, Saint Joseph, MI, USA). Drilling cuttings were obtained from both the 1/2 radius and the center at the same height: one for Ce, Si, Mn, P, Cr, Ni, and Mo analysis by inductively coupled plasma emission spectrometry (ICP–AES,710–ES, Agilent Technologies, Santa Clara, CA, USA) and the other for C and S analysis by infrared absorption (CS–3000, NCS, Beijing, China). The measured main compositions for different samples after Ce addition are shown in Table 2. Another cylinder-shaped sample with a height of 10 mm was cut from the position 40 mm away the bottom of the ingot. After polishing, the surface was etched with an HCl water solution (volume fraction 1:1) at 80 °C for 15 min, and then the equiaxed ratio was calculated using the image processing software Image Pro Plus 6.0. Subsequently, three metallographic samples (No. 1–3) with the dimensions of 8 mm × 8 mm × 10 mm were cut along the diameter of the cylinder-shaped sample, as shown in Figure 2. After grinding and polishing, the morphology of typical non-metallic inclusions in the No. 3 sample was first observed using a Phenom Pro SEM, and their chemical compositions were analyzed by an energy spectrum analyzer (EDS) (Carl Zeiss, Oberkochen, Germany). A total of 20 pictures under a magnification of 2000–3000 times for each sample were taken by the SEM. The total number of inclusions was summed, and then the size distribution of inclusions was counted and analyzed using the Image Pro Plus 6.0 software. Additionally, the formation of inclusions was calculated using classic thermodynamic methods and Factsage software 8.1. Subsequently, heat treatment experiments were carried out on the other two metallographic samples (No. 1 and 2). As shown in Figure 3a, the No. 1 sample was heated to 890 °C in the furnace, held for 30 min, and then water-quenched. The sample was etched with a saturated picric acid solution at 70 °C for 1 min after grinding and polishing, and the secondary dendrite arm spacing was measured using an optical microscope. The No. 2 sample was put in a furnace at 890 °C, held for 6 min, and then cooled to room temperature with the furnace (Figure 3b). It was etched with 4% nitrate alcohol for 8 s after grinding and polishing, and the pearlite lamella spacing was observed and calculated using a ZEISSEVO18 SEM (Carl Zeiss, Oberkochen, Germany).

## 3. Results and Discussion

The content and yield of the measured Ce in drilling samples are listed in Table 1. The contents in the T1–T3 schemes were 0.011, 0.017, and 0.075 wt%, and the yields were 27.6%, 41.5%, and 90.4%, respectively. Although a similar amount of Ce was added to the molten steel in the T1 and T2 schemes, the yield in T2 was higher due to the deeper Ce–Fe addition location and stronger stirring compared to the T1 scheme. Therefore, this indicates that the addition method of RE directly influences its yield. This is also one of the reasons why unstable RE yield frequently occurs in industrial applications.

### 3.1. Effect of Ce Addition on Oxygen and Sulfur Content in Steel

**Figure 4:**
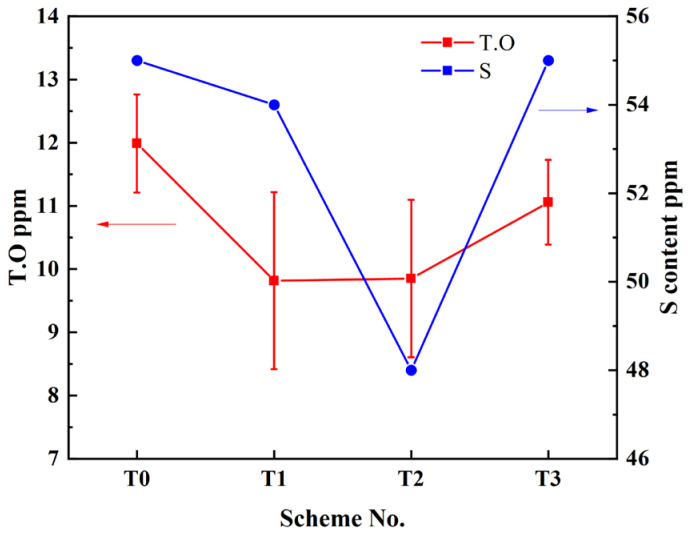
Total oxygen and sulfur contents in steel across different schemes.

It is well known that the content of total oxygen (T.O.) and sulfur reflects the cleanliness of molten steel. In order to study the purification effect of RE, the contents of T.O. and S in different schemes were chemically measured, and the results are shown in Figure 4.

It was observed that with the increase in Ce content from T0 to T3, the oxygen and sulfur contents both decreased initially and then increased. The T.O. content in the T1 scheme (Ce 0.011 wt%) was 9.82 ppm, demonstrating a decrease of 2.17 ppm compared with that in the T0 scheme without Ce addition. In contrast, in the T3 scheme, the T.O. increased to 11.05 ppm, with Ce content increasing to 0.075 wt%. The S content in the T2 scheme was the lowest, being 7 ppm lower than that in T0. However, the S contents in the T1 and T3 schemes were almost the same. This indicates that only an appropriate amount of RE can positively affect the purification of molten steel, consistent with the result in reference [20]. When an appropriate amount of RE is added, it can react with O and S to form rare earth oxide and sulfide inclusions, which can be removed by floating to the slag. As a result, the O and S contents in molten steel decrease. However, when an excessive amount of RE is added to the steel, the number of RE inclusions will increase significantly, and the removal efficiency is lowered due to their high density.

### 3.2. Effect of Ce Content on the Characteristics of Inclusions in Steel

**Figure 5:**
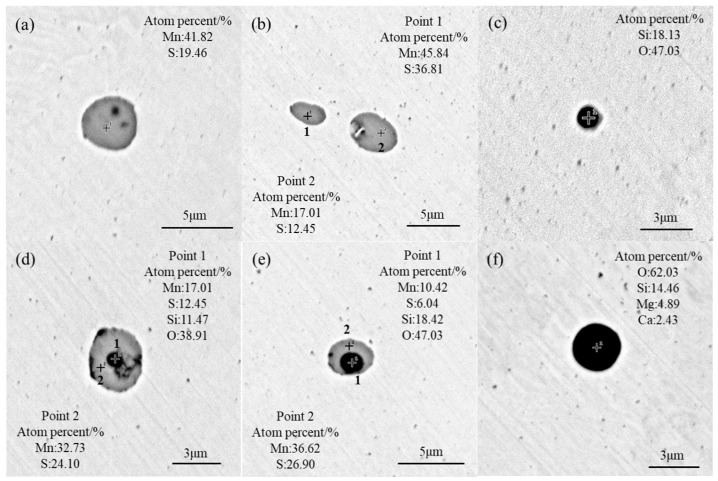
Morphology and composition of typical inclusions in the T0 scheme: (**a**,**b**) MnS; (**c**) SiO_2_; (**d**,**e**) SiO_2_ + MnS; (**f**) CaO–SiO_2_–MgO inclusions.

Figure 5 shows the morphology and composition of typical inclusions in the sample from the T0 scheme. The main inclusions were MnS, SiO_2_, SiO_2_–MnS, and CaO–SiO_2_–MgO. The MnS inclusions appeared mostly as spherical or ellipsoidal shapes in grey color, with sizes ranging from 1 to 3 μm (Figure 5a,b), although some of them were larger than 8 μm. The SiO_2_ inclusions were spherical and black, with sizes smaller than 3 μm (Figure 5c); they formed in the steelmaking process due to the adoption of Si–Mn deoxidizer in industrial production. During the solidification of molten steel, MnS nucleated with the substrate SiO_2_ and then grew into MnS–SiO_2_ composite inclusions (as shown in Figure 5d,e). The CaO and MgO inclusions were generally from slag and/or furnace refractory, then reacted with SiO_2_ to form CaO–SiO_2_–MgO complex inclusions with sizes around 3 μm (Figure 5f).

**Figure 6:**
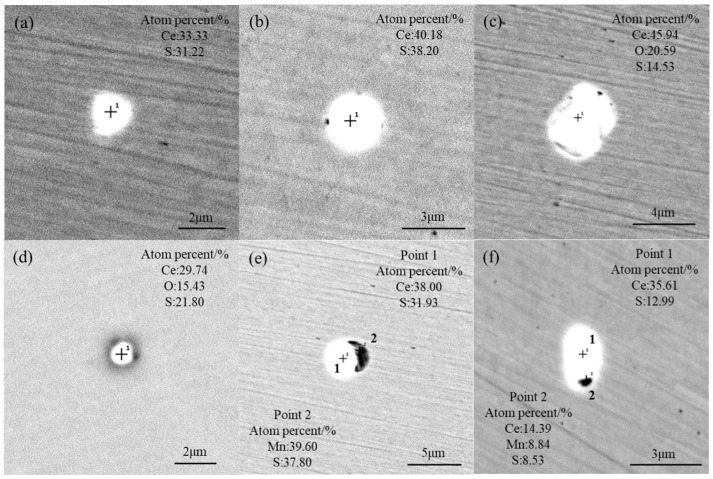
Morphology and composition of typical inclusions in the T1 scheme: (**a**,**b**) CeS; (**c**,**d**) Ce–O–S; (**e**,**f**) Ce–S + MnS.

After adding 0.040 wt% Ce to the T1 scheme, Ce reacted with S and O to generate a large amount of Ce–S (Figure 6a,b) and Ce–O–S inclusions (Figure 6c,d), and it modified MnS into Ce–S + MnS type inclusions (Figure 6e,f). These inclusions were mainly spherical and ellipsoidal in shape, with sizes less than 3 μm. Compared with the T0 scheme, the size of inclusions in the T1 scheme was reduced as the fraction of small-sized inclusions increased. Pure MnS inclusions were not observed in T1 but appeared in large amounts in T0, indicating that the MnS inclusions in T1 have been modified into Ce–S or Ce–S + MnS (Ce–Mn–S) inclusions by Ce. The reason will be explained by a thermodynamics calculation in the following text.

**Figure 7:**
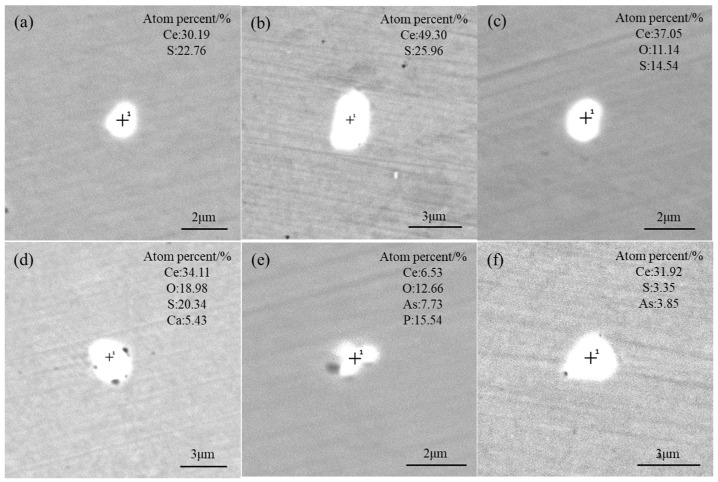
Morphology and composition of typical inclusions in the T2 scheme: (**a**,**b**) Ce–S; (**c**) Ce–O–S; (**d**) Ce–O–S–Ca; (**e**) Ce–O–P–As; (**f**) Ce–S–As.

As shown in Figure 7a–c, the inclusions in the T2 scheme were still predominated by Ce–S and Ce–O–S, with their sizes less than 3 μm. However, the aspect ratios of inclusions tended to increase, and the number of ellipsoidal Ce-containing inclusions also increased. In addition to modifying MnS into Ce–S inclusions, Ce also reacted with CaO–SiO_2_–MgO composite inclusions to generate Ce–O–S–Ca inclusions, as shown in Figure 7d. According to the Ellingham diagram [13], the deoxidation ability of RE is higher than that of Si and Mg. Therefore, the Si and Mg elements in CaO–SiO_2_–MgO composite inclusions were replaced by Ce, and then Ce–O–S–Ca inclusions finally formed with the combination of S.

Although the designed amount of Ce added by Fe–Ce–Si alloy in the T2 scheme was only 0.001 wt% higher than that in the T1 scheme, the Ce content reached 0.017 wt% due to a deeper addition location of the RE master alloy into the bath and stronger stirring compared with T1. Consequently, the type of inclusions in this scheme changed significantly. As shown in Figure 7e,f, a small number of rare earth inclusions containing As and P started to precipitate. Since P and As have a lower affinity for RE elements compared to S and O, this indicates that the content of Ce is adequate in T2. The appearance of Ce–O–P–As and Ce–S–As inclusions was unexpected. In addition, only a very small amount of MnS inclusions were incompletely modified in T1, suggesting that the Ce content in that scheme was close to the critical value of full modification of sulfides.

**Figure 8:**
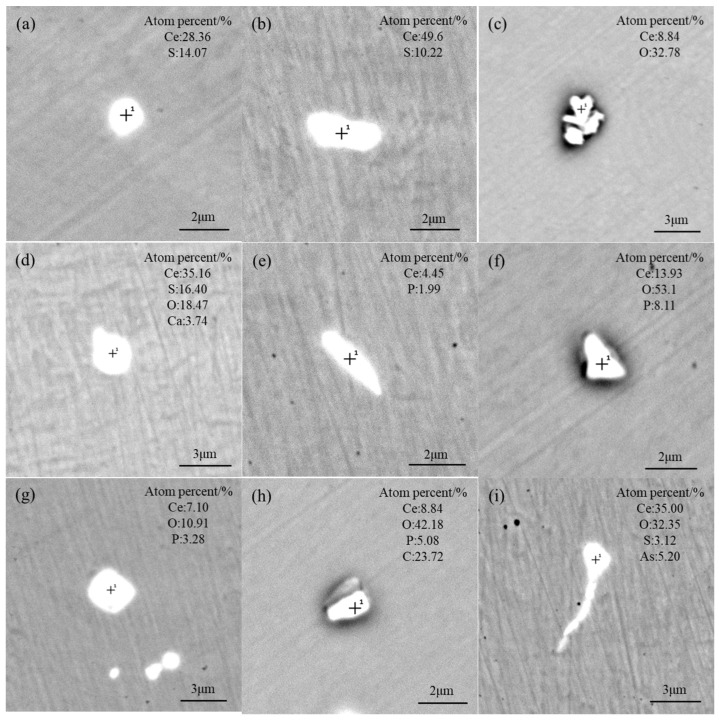
Morphology and composition of typical inclusions in the T3 scheme: (**a**,**b**) Ce–S; (**c**) Ce–O; (**d**) Ce–O–S–Ca; (**e**) Ce–P; (**f**,**g**) Ce–O–P; (**h**) Ce–O–P–C; (**i**) Ce–O–S–As.

The designed Ce addition amount in the T3 scheme is about twice as high as in T2, and the final content is the highest. As shown in Figure 8, the MnS inclusions completely disappeared, as they were completely modified into Ce–S; both the length and width were larger than that in the T1 and T2 schemes. In addition, a large number of RE inclusions containing As and P appeared, with morphologies mostly in long strips and angular shapes. In the T3 scheme, the cluster-like Ce–O inclusions were also detected, with a size of approximately 3 μm, as shown in Figure 8c. The Ce–O–S–Ca inclusions still existed in the steel (Figure 8d), indicating that Ce cannot completely reduce Ca under the present conditions. After modifying MnS and CaO–SiO_2_–MgO inclusions, the excessive Ce reacted with P to form Ce–P type inclusions and with O and C to generate Ce–O–P and Ce–O–P–C, as shown in Figure 8f–h. The size of Ce–P and Ce–O–P–C inclusions were relatively large and generally in a long strip shape. The Ce–O–P inclusions were in triangular and quadrilateral shapes, with sharp edges and angles, which can easily cause stress concentration. As seen in Figure 8i, Ce combined with O, S, and As to form Ce–O–S–As inclusions with tadpole-like shapes, which could be harmful to the mechanical properties in the transverse direction. As a result, the addition of RE should not be excessive; otherwise, the size and shape of inclusions will be unfavorable.

### 3.3. Thermodynamics of Inclusion Formation

In order to explain the formation of the aforementioned inclusions, the thermodynamics in molten steel was calculated. The standard Gibbs free energy expression of the relevant reactions and the values at 1873 K are shown in Table 3. The universal chemical reaction equation is expressed as Equation (1), and the Gibbs free energy is calculated using Formula (2).

(1) A+xyB=1xAxBy

(2) ΔG=ΔGθ+RTlnJ=ΔGθ+RTlna1xAxByaA×aBxy

where *A* and *B* represent the reactants; $A_{x}B_{y}$ is the reaction product by *A* and *B*; ΔG is the Gibbs free energy of the reaction (J·mol^−1^); $\Delta G^{\theta }$ is the standard Gibbs free energy for the reaction (J·mol^−1^); *R* is the ideal gas constant (8.314 J·mol^−1^·K^−1^); *J* is the activity ratio of the product to the reactant; $a_{i}$ is the activity of reactant or product *i*. In this study, the reactant *A* in Equation (1) mainly refers to the Ce element, while *B* represents other reactants listed in Table 3.

The data for CeP are obtained from different references through a five-step calculation [24,25,26]. The activity coefficient of component *i* was calculated using Equation (3) [27]:

(3) $$lgf_{i}=\overset{n}{\underset{j}{\sum }}e_{i}^{j}w{\left[j\right]}_{\%}$$

Taking a 1% concentration as the standard state, the activity of component i can be expressed as follows:

(4) $$a_{i}=f_{i}\cdot w{\left[i\right]}_{\%}$$

where $f_{i}$ is the activity coefficient of element i; $e_{i}^{j}$ is the interaction coefficient of element j on i; $w{\left[i\right]}_{\%}$ is the weight fraction of element i, %.

Table 4 presents the interaction coefficients among various elements [10,11,12] in 55SiCr spring steel at 1873 K, where a “–” represents that no interaction coefficient is available and thus considered zero in this study. The interaction coefficients were substituted into Formulas (3) and (4) to obtain the activities of Ce, O, S, and Mn at different Ce contents, as shown in Table 5. These activities were substituted into Formulas (1) and (2) to calculate the Gibbs free energy of the relevant reactions in Table 3, with the results shown in Table 6.

It can be seen from Table 6 that, for the Ce contents in the T1–T3 schemes, the Gibbs free energy values for the formation of MnS in both solid or liquid states at 1873 K were all positive, which indicates that MnS inclusions cannot form during the steelmaking process at 1873 K. The MnS inclusions observed in the experiment likely originated from the base material. Analysis of the base material revealed that the fraction of MnS inclusions in the matrix was 82%. Since the melting temperature of MnS is 1893 K, it cannot be fully dissolved into the molten steel at the experimental temperature of 1873 K, resulting in most of it remaining in the molten steel. Upon the addition of the Ce alloy, these inclusions were first modified into Ce–Mn–S and finally into Ce–S.

Table 6 also shows that the ΔG of the reactions (1)–(9) were all negative at 1873 K, indicating that all types of RE inclusions could potentially form under the present conditions. The ΔG values of reactions (7)–(9) were lower than those for other reactions, implying that Ce will first modify MnS inclusions after being added into the molten steel. Subsequently, it reacts with O and/or S to form Ce–O, Ce–S, and/or Ce–O–S inclusions, as observed in Figure 6, Figure 7 and Figure 8. For Ce–O type inclusions, Ce_2_O_3_ is more likely to form than CeO_2_, as the ΔG for Ce_2_O_3_ formation is much smaller.

**Figure 9:**
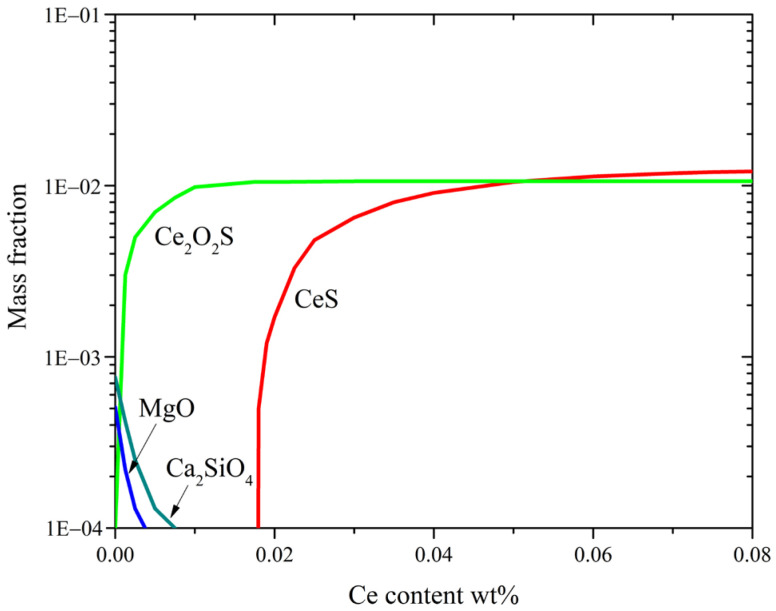
Variation of different inclusions against Ce content at 1873 K.

In addition, Ce can reduce MgO, SiO_2,_ and CaO compounds in liquid steel, as shown in Figure 7 and Figure 8. The MnS + CaO–SiO_2_–MgO inclusions are modified into the Ce–O–Ca–S system. The thermodynamic diagram illustrating the variation of different inclusions with respect to Ce content at 1873 K, generated using the Factsage software, is shown in Figure 9. It is evident that the original MgO and Ca_2_SiO_4_ compounds can react with Ce, but they disappear as Ce content increases to approximately 0.01%. However, due to kinetic constraints, the reaction cannot undergo completion during the experiment, resulting in the retention of some Ca-containing complex oxides.

For the formation of Ce–P–O and Ce–As–S inclusions, the Gibbs free energy of CeP is provided in Table 6, while no thermodynamic data has been reported for CeAs. It can be seen that the ΔG value of CeP is negative, indicating that CeP can exist in liquid steel. However, this value is higher than that of CeS, Ce_2_O_3,_ and Ce_2_SO_2_, suggesting CeP inclusions are less stable. The activity of Ce in the T2 and T3 schemes is significantly higher than in T1, leading to a notable decrease in ΔG values and an increased likelihood of formation. This result is in good agreement with experimental observations. In addition, the appearance of CeP and CeAs is also closely related to the strong segregation of Ce, P, and As during solidification, especially in samples with high alloying content [28]. These inclusions have also been reported in many steels [29,30,31,32]. In fact, the formation of CeP and CeAs can reduce the harmful effects of P and As segregation at grain boundaries, but the size of inclusions should not be too large [33]. Otherwise, the fatigue properties and impact toughness may deteriorate. Overall, the thermodynamic results are generally consistent with the energy spectrum analysis of the inclusions in T1–T3 schemes.

### 3.4. Effect of Ce Content on the Number and Size of Inclusions

**Figure 10:**
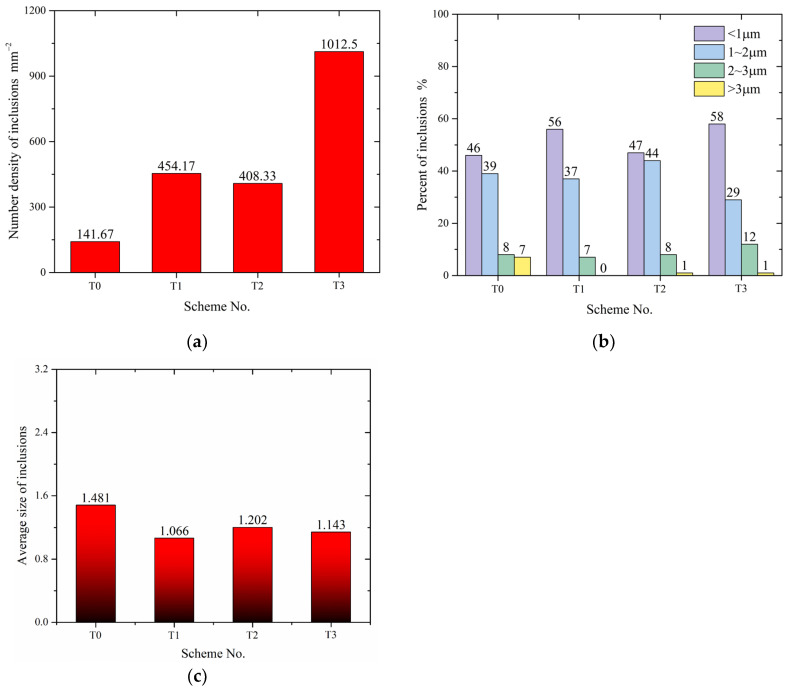
Comparison of characteristics of inclusions. (**a**) Number density; (**b**) size distribution; (**c**) average size.

Figure 10 shows the characteristics of inclusions in the No. 3 samples from T0–T3 schemes. In Figure 10a, the number density of inclusions is presented as the ratio of the total number of inclusions counted in the view field to the area of that field. Figure 10b shows the proportion of inclusions of different sizes, obtained by calculating the ratio of the number of inclusions within a specific size range, such as 1–2 μm, to the total number of inclusions across all sizes. In Figure 10c, the total size of all inclusions detected in the view fields was computed and then divided by the total number of inclusions to obtain the average size.

When the Ce content was 0.011 wt% (T1), the number density of inclusions in steel increased from 141.67 #/mm^2^ without Ce addition (T0) to 454.17 #/mm^2^, while the average size of inclusions decreased from 1.481 μm to 1.066 μm. The proportion of the inclusions with a diameter smaller than 1 μm increased from 46% to 56%, and the inclusions larger than 3 μm essentially disappeared. With a further increase in the Ce content to 0.017%, the number density of inclusions in the T2 scheme decreased slightly, while the size of inclusions increased to 1.202 μm. The proportion of inclusions with sizes between 1–3 μm in T2 increased compared to T1, while those below 1 μm decreased. Combining with the characteristics of the inclusions in the T2 scheme, it is believed that the amount of Ce in this sample was slightly higher than required for the full modification of MnS inclusions, causing the RE inclusions to aggregate and presenting a slight increase in size and a decrease in number density. In the T3 scheme, a large number of small-sized, irregular-shaped inclusions appeared, and the proportion of inclusions smaller than 1 μm was the highest, making the number density of inclusions seven times higher than that in the T0 scheme. The average grain size decreased to 1.143 μm, mainly attributed to the increase in small-sized inclusions. As stated above, Ce–P, Ce–O–P, Ce–O–S–As, and Ce–O–P–As-type inclusions were generated abundantly in this scheme. This is the explanation for the dramatic increase in number density. References report that an appropriate quantity of inclusions sized 1–3 μm can refine the solidification structure of steel [34], but an excessive amount, especially of large particles, can lead to the deterioration of the microstructure and performance [35]. The proportion of inclusions with a diameter larger than 2 μm increased in the T3 scheme, suggesting notable aggregation occurred before solidification. The variation in inclusion size reveals that an appropriate amount of Ce addition to steel helps adjust large-sized inclusions like MnS; however, an excessive amount can result in deterioration. From the perspective of inclusion and size control, it is believed that the amount of Ce added in the T1 and T2 schemes appears reasonable in the present situation.

### 3.5. Effect of Ce Content on the Solidification Structure

**Figure 11:**
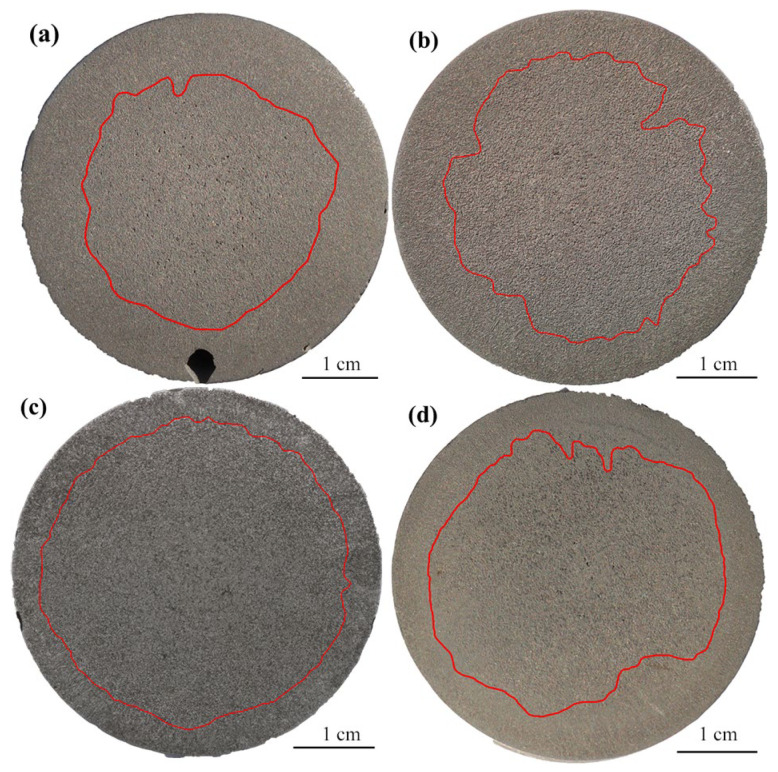
The solidification structure and CET of each scheme: (**a**) T0; (**b**) T1; (**c**) T2; (**d**) T3.

**Figure 12:**
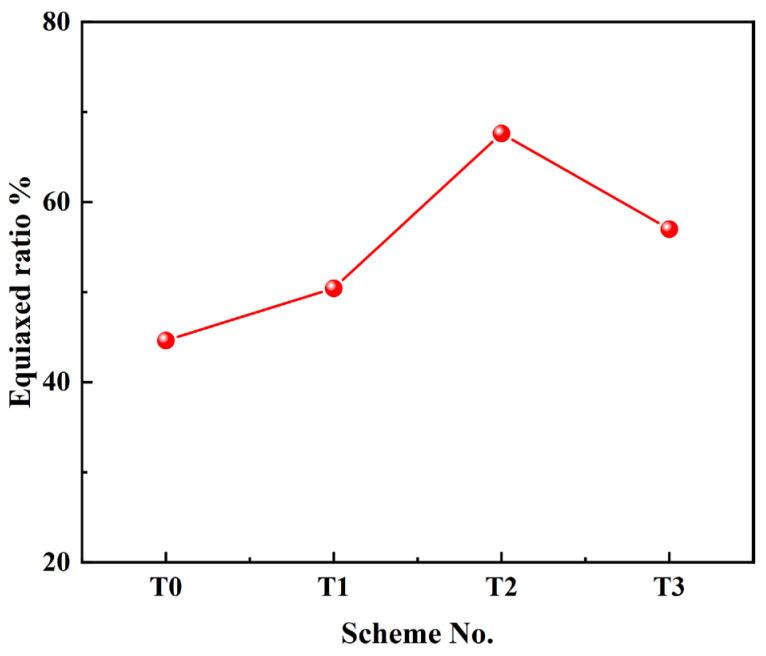
Comparison of equiaxed ratio in different schemes.

The influence of Ce content in the T0–T3 schemes on the solidification structure of 55SiCr steel is shown in Figure 11, where the red solid line marks the interface of the columnar to equiaxed transition (CET). Outside the red line is the columnar dendrite zone, with primary grains showing an obvious orientation and a long axis roughly perpendicular to the mold wall. Inside the red line is the equiaxed dendrite zone, where the grains show no obvious orientation and have a more uniform property distribution [23]. The equiaxed ratio was calculated as the area of the equiaxed dendrite zone relative to the total area in Figure 11, using Image Pro Plus 6.0 software; the results are shown in Figure 12. The equiaxed ratio in the solidification structure in the T0 scheme was about 44.63%. With the addition of Ce, the equiaxed zone expanded. When the content of Ce was 0.011 wt% (T1), the equiaxed ratio increased to 50.42%, suggesting that the nucleation of equiaxed dendrite was promoted. When Ce content further increased to 0.017 wt% (T2), the equiaxed ratio reached a maximum value of 67.63%, a clear increase of 23% compared with the T0 scheme. However, when Ce content increased to 0.075 wt%, the equiaxed ratio decreased to 56.99%, indicating the nucleation stimulation effect decreased unexpectedly.

The above results show that rare earth addition has a significant effect on the solidification structure evolution in 55SiCr spring steel. Many studies [10,11,23,36] have shown that heterogeneous nucleation is the dominant mechanism for the expansion of the equiaxed zone in steel. According to lattice misfit theory, two requirements need to be satisfied for particles to act as the core of heterogeneous nucleation. First, the melting point of nucleation particles should be higher than the liquidus temperature of steel so a solid substrate can be referenced. Second, there should be a low misfit between the nucleation core and the primary solidification phase during steel solidification. According to previous investigations [23,36], the substrate has a good heterogeneous nucleation effect when the lattice misfit is less than 6%. A promotional impact may occur when the misfit is between 6% and 12%. However, substrates with a misfit larger than 12% cannot serve as a nucleation core.

When Ce was added to molten steel, a large number of Ce_2_O_3_, Ce_2_O_2_S, and CeS were generated. The corresponding melting points of these compounds are all above the liquidus temperature of 55SiCr steel, as shown in Table 7. The lattice misfit of Ce_2_O_3_, Ce_2_O_2_S, and CeS with ferrite is less than 6% [7], hence, the capability to stimulate heterogeneous nucleation is favorable. However, the misfit between CeP or CeAs and ferrite is calculated to be higher than 20% for (100)_CeP/CeAs_//(100)_δ–Fe_, (100)_CeP/CeAs_//(110)_δ–Fe_, and (100)_CeP/CeAs_//(111)_δ–Fe_ based on the lattice parameters from reference [37,38], suggesting a low potential for heterogeneous solidification. The generation of particles with a low misfit in molten steel reduces the undercooling for nucleation, leading to the early formation of CET during solidification. As a result, the equiaxed zone expanded and the equiaxed ratio increased. In addition, dissolved Ce concentrated at the dendrite interface can hinder grain growth and refine the solidification structure.

With the further increase in Ce content to 0.075 wt%, the proportion of equiaxed crystals decreased. The excessive increase in rare earth content resulted in a high amount of Ce–P, Ce–As, Ce–O–P, Ce–O–P–As, and Ce–O–S–As inclusions, with misfit to the primary ferrite phase much larger than 12%. Consequently, the stimulation effect on heterogeneous nucleation decreased accordingly. In previous studies [39,40], it was found that CeP is likely to nucleate on attached Ce–S or Ce–O–S inclusions, preventing the heterogeneous solidification by low misfit particles. In addition, there is also a tendency for the inclusions to aggregate as the content of Ce increases from 0.017% (T2) to 0.075% (T3), which is also not favorable for heterogeneous nucleation potential. From the perspective of solidification structure evolution, the suitable addition amount of Ce is between the T1 and T2 schemes.

### 3.6. Effect of Rare Earth Content on Secondary Dendrite Arm Spacing

**Figure 13:**
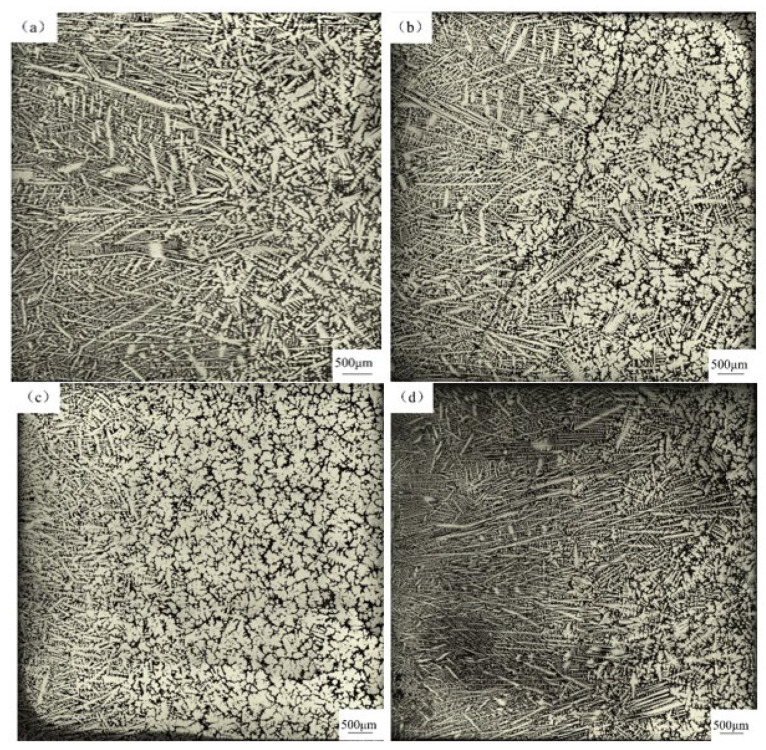
Dendrite morphology in different schemes. (**a**) T0; (**b**) T1; (**c**) T2; (**d**) T3.

**Figure 14:**
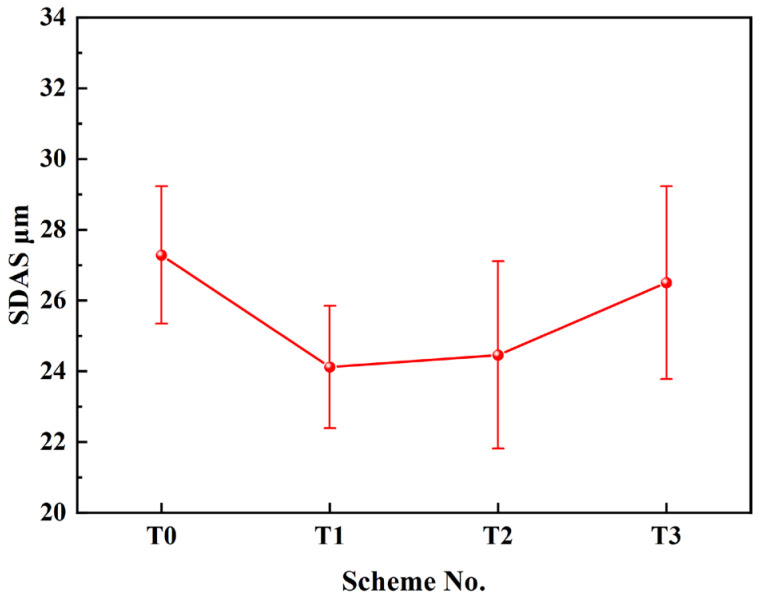
Statistical results of SDAS at different schemes.

The secondary dendrite arm spacing (SDAS) in steel typically reflects its solidification properties, such as microshrinkage, microsegregation, and the distribution homogeneity of precipitates. A smaller SDAS usually corresponds to less element segregation in the as-cast matrix, which is favorable for improving the homogeneity of the final product. Figure 13 shows the dendrite morphology of the No. 1 sample in different schemes. The location of the CET can be easily identified, and the results are consistent with the data in Figure 11. Additionally, the dendrite coarsening behavior in different schemes is also compared. The equiaxed dendrite is well-developed in the T0 scheme, and the contour is sufficiently sharp. However, the equiaxed dendrite is refined in the T1 and T2 schemes, as the morphology becomes more globular and the diameter becomes smaller. In the T3 scheme, the shape of the equiaxed dendrite sharpens again, and the overall size increases. Semi-macrosegregation generally occurs in the enclosure zone between coarse dendrites; therefore, the SDAS was measured in the equiaxed zone. Ten view fields were selected for each scheme, and a total of 100–150 SDASs were measured from 8 to 10 different primary dendrites using Image Pro Plus 6.0 software. The average value was then calculated, as shown in Figure 14. It can be seen that the average SDAS was 27.28 μm for the T0 scheme without Ce addition, while it decreased to 24.12 μm as the Ce content increased to 0.011%, which is about 11.6% lower than the original, indicating a significant refinement in the solidification microstructure. The Ce content in the T2 scheme was higher than that in T1, and the average SDAS was slightly increased by 0.34 μm. The T3 scheme had the highest Ce content of 0.075 wt%, and the SDAS increased to 26.5 μm, still showing some refinement compared to the T0 scheme. The refining of SDAS is actually the result of a decrease in dendrite size, which can be easily observed in Figure 13. Small equiaxed dendrites correspond to a short time for dendrite arm growth and coalescence, preventing significant development. This is the dominant mechanism for the prevention of dendrite coarsening. In addition, Ce readily enriches in the interdendritic zone due to the strongly selective crystallization, and the constitutional undercooling in the liquid increases, leading to an increase in interface instability and the branching of dendrite arms [41]. However, this effect is not quite obvious, as the average concentration is too low. The weak refining impact of dissolved Ce on SDAS has been discussed in our previous study [9]. From the perspective of solidification microstructure refinement, the addition amount of Ce between the T1 and T2 schemes should be appropriate.

### 3.7. Effect of Rare Earth Content on the Pearlite Lamellar Spacing

**Figure 15:**
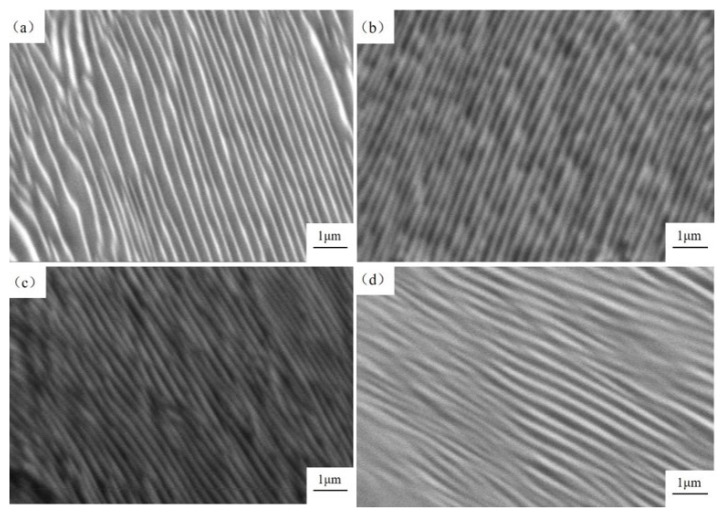
Morphology of pearlite lamellar of sample No. 2 at different schemes: (**a**) T0; (**b**) T1; (**c**) T2; (**d**) T3.

**Figure 16:**
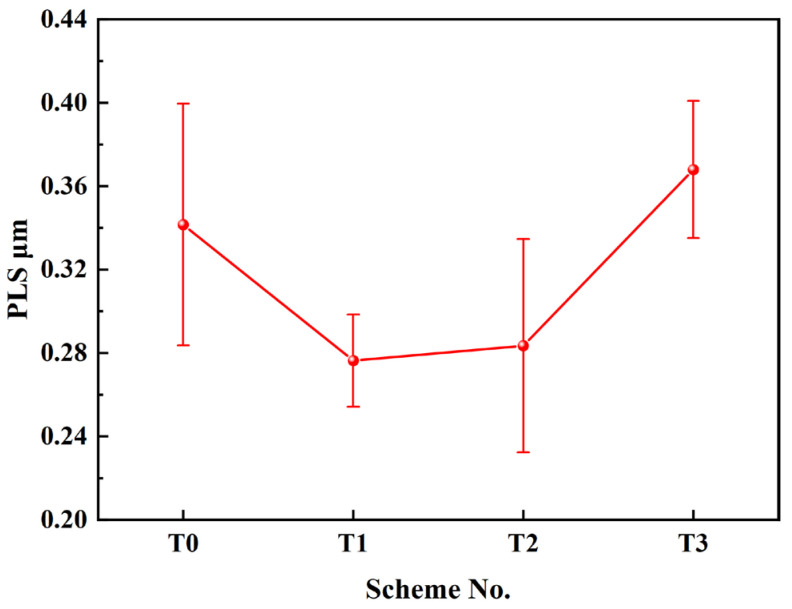
Pearlite lamellar spacing for different schemes.

The refinement of pearlite lamellar spacing (PLS) can enhance the comprehensive mechanical properties of steel products. When PLS is reduced, the phase interface in the microstructure increases, which in turn increases the resistance to plastic deformation accordingly. Meanwhile, a thin cementite sheet is easy to deform under external load, improving the plasticity and toughness of steel [42]. Figure 15 shows the pearlite morphology in sample No. 2 by SEM from different schemes. It is observed that the PLS decreases initially from T0 to T1 and T2, then increases in T3, corresponding to the SDAS results in Figure 12 and Figure 13. To obtain the value of PLS, ten view fields were selected for each scheme, and seven to ten complete and uniform pearlite lamellae were picked from each view. The spacing of the pearlite lamellae was measured using Image Pro Plus 6.0 software, and the average value was calculated, as shown in Figure 16.

It is seen that the average PLS was 0.342 μm in the T0 scheme without Ce addition. It was then refined to 0.276 μm when the Ce content reached 0.011% (T1), decreasing by an appropriate 19.3%. With the continuous increase in Ce content, the average PLS increased to 0.283 μm in the T2 scheme, meaning that the refinement effect of Ce was impaired compared with the T1 scheme. When the Ce content increased further to 0.075%, the average PLS reached 0.368 μm, which was even larger than that in the T0 scheme. In addition, the pearlite lamellar morphology in the T3 scheme was abnormal compared to the others, as the orientation was more disordered. The addition of Ce can reduce the diffusion coefficient of carbon atoms in austenite, thus postponing the nucleation and growth of cementite to a lower temperature range with significant undercooling, which refines the pearlite microstructure [43]. Meanwhile, the dissolved Ce will segregate along the austenite grain boundary and decrease the content of other elements in this area, leading to an increase in the activation energy and a delay in the occurrence of pearlite transformation [43]. These should be the explanations for the thinning of PLS using a proper amount of Ce alloying. However, when Ce content is excessive, the refining effect on PLS is diminished or even becomes negative. There will be numerous Ce inclusions in the austenite matrix acting as nucleation sites for new phases, allowing pearlite transformation to occur earlier at high temperatures. The diffusion ability is higher in this scenario, resulting in a larger PLS. The disordered orientation in the T3 scheme can be attributed to the increased number of nucleation sites. In addition, a high amount of dissolved Ce reduces the interface stability, promoting the branching of ferrite or cementite lamellae. In order to obtain fine pearlite lamellae and outstanding properties, a reasonable amount of Ce should be between the T1 and T2 schemes.

## 4. Conclusions

(1) An appropriate amount of Ce addition can effectively modify the non-metallic inclusions in 55SiCr spring steel and refine the solidification structure, including a reduction in equiaxed dendrite size, SDAS, and PLS. The optimum Ce content is 0.011–0.017 wt% for the present study.
(2) The rare earth element Ce shows a purifying effect on 55SiCr spring steel. When the content reaches 0.017 wt%, the total oxygen content in the matrix decreases from 11.99 ppm in the Ce-free scheme to 9.95 ppm, and the sulfur content decreases from 55 ppm to 48 ppm.
(3) MnS inclusions are almost completely modified into spherical and small-sized Ce–S and Ce–O–S inclusions when the Ce content is about 0.017 wt%. At the same time, small-sized Ce–P and Ce–O–P–C inclusions begin to appear. As the Ce content increases to 0.075 wt%, large amounts of Ce–P and Ce–P–O inclusions are generated. The formation mechanism of the different Ce-containing inclusions is well explained by Gibbs free energy calculations and the thermodynamic diagram.
(4) With the increase in Ce content in 55SiCr spring steel, the equiaxed ratio in the solidification structure initially increases and then decreases, while the variations of SDAS and PLS present the opposite trend. The largest equiaxed ratio occurs at 0.017 wt% Ce content, and the smallest SDAS and PLS are observed at 0.011 wt% Ce.

## Figures and Tables

**Table 1:** Ce alloying scheme in vacuum induction furnace.

Case	Mass of Steel Sample g	Ce Theoretical Addition wt%	Actual Tested Ce Content wt%	Ce Yield %
T0	1302.83	0	0	
T1	1350.8	0.040	0.011	27.6
T2	1243.4	0.041	0.017	41.5
T3	1258.3	0.083	0.075	90.4

**Table 2:** Chemical composition of spring steel raw materials (wt%).

	C	Si	Mn	P	S	Cr	O	N	Ni	Mo	Ce
T0	0.56	1.45	0.68	0.0094	0.0055	0.71	0.0012	0.0038	0.01	0.01	0
T1	0.55	1.46	0.67	0.0092	0.0054	0.69	0.0010	0.0039	0.01	0.01	0.011
T2	0.56	1.47	0.68	0.0091	0.0048	0.70	0.0010	0.0036	0.01	0.01	0.017
T3	0.56	1.46	0.67	0.0091	0.0055	0.70	0.0011	0.0040	0.01	0.01	0.075

**Table 3:** Reactions in molten steel and standard reaction Gibbs free energy at 1873 K [21,22,23].

Reaction	Δ*G^θ^*/J·mol^−1^	Δ*G^θ^* Value /J·mol^−1^
[Ce] + 2[O] = CeO_2_(s)	−854,270 + 250*T*	−386,020
[Ce] + 3/2[O] = 1/2Ce_2_O_3_(s)	−715,560 + 180*T*	−378,420
[Ce] + [O] + 1/2[S] = 1/2Ce_2_O_2_S(s)	−676,505 + 164*T*	−366,149
[Ce] + [S] = CeS(s)	−422,780 + 121*T*	−196,147
[Ce] + 3/2[S] = 1/2Ce_2_S_3_(s)	−537,290 + 164*T*	−230,118
[Ce] + 4/3[S] = 1/3Ce_3_S_4_(s)	−498,480 + 146.3*T*	−224,460
[Ce] + [P] = CeP(s)	−420,073 + 143.29*T*	–151,691
[Mn] + [S] = MnS(l)	−131,624 + 79.07*T*	16,474
[Mn] + [S] = MnS(s)	−158,365 + 93.996*T*	17,689
[Ce] + MnS(s) = CeS(s) + [Mn]	−264,415 + 27.004*T*	−213,837
[Ce] + 3/2MnS(s) = 1/2Ce_2_S_3_(s) + 3/2[Mn]	−299,742.5 + 23.006*T*	−256,652
[Ce] + 4/3MnS(s) = 1/3Ce_3_S_4_(s) + 4/3[Mn]	−287,326.7 + 20.972*T*	−248,046

**Table 4:** Interaction coefficients between elements in steel at 1873 K.

	C	Si	Mn	P	S	Cr
S	0.11	0.063	−0.026	0.029	−0.046	−0.011
Mn	−0.07	−0.0327	0	−0.0035	−0.048	0.0039
Ce	−0.077	–	0.13	1.77	−39.8	–
P	0.13	0.12	0	0.062	−0.028	−0.03
O	−0.45	−0.131	−0.021	0.07	−0.133	−0.04
	Al	V	O	N	Ca	Mg
S	0.035	−0.016	−0.27	0.01	−110	−1.82
Mn	0.07	0.0057	−0.083	−0.091	−0.023	–
Ce	−2.25	−0.33	−5.03	−6.599	–	–
P	–	–	0.13	0.094	–	–
O	−3.9	−0.3	−0.2	0.057	−271	–
	Ti	Mo	B	Ni	Cu	Ce
S	−0.18	0.0027	0.13	0	−0.0084	−0.856
Mn	−0.05	0.0045	0.22	−0.0071	–	0.054
Ce	−3.62	–	–	–	−0.486	−0.003
P	–	–	–	0.0002	0.024	–
O	−0.6	0.0035	−2.6	0.006	−0.013	−0.57

**Table 5:** Activities of elements under different Ce contents at 1873 K.

Scheme	*a_i_*				
	*a*_Ce_	*a*_O_	*a*_s_	*a*_Mn_	*a_P_*
T1 (0.011%Ce)	7.02 × 10^−3^	2.59 × 10^−4^	6.57 × 10^−3^	0.5518	0.01517
T2 (0.017% Ce)	1.15 × 10^−2^	2.57 × 10^−4^	5.77 × 10^−3^	0.5522	0.01521
T3 (0.075% Ce)	4.74 × 10^−2^	2.68 × 10^−4^	5.90 × 10^−3^	0.5562	0.01526

**Table 6:** Formation Gibbs free energy of rare earth inclusions in molten steel at 1873 K.

Reaction	No.	Δ*G* J·mol^−1^		
		T1	T2	T3
[Ce] + 2[O] = CeO_2_(s)	(1)	−51,545	−59,035	−82,322
[Ce] + 3/2[O] = 1/2Ce_2_O_3_(s)	(2)	−108,260	−115,786	−138,773
[Ce] + [O] + 1/2[S] = 1/2Ce_2_O_2_S(s)	(3)	−121,178	−127,731	−150,586
[Ce] + [S] = CeS(s)	(4)	−40,679	−46,296	−68,720
[Ce] + 3/2[S] = 1/2Ce_2_S_3_(s)	(5)	−35,524	−40,132	−62,724
[Ce] + 4/3[S] = 1/3Ce_3_S_4_(s)	(6)	−42,908	−47,852	−70,388
[Ce] + MnS(s) = CeS(s) + [Mn]	(7)	−145,881	−153,502	−175,478
[Ce] + 3/2MnS(s) = 1/2Ce_2_S_3_(s) + 3/2[Mn]	(8)	−193,326	−200,941	−222,862
[Ce] + 4/3MnS(s) = 1/3Ce_3_S_4_(s) + 4/3[Mn]	(9)	−183,177	−190,794	−212,733
[Ce] + [P] = CeP(s)	(10)	−9246	−16,973	−39,079
[Mn] + [S] = MnS(l)	(11)	25,733	25,721	25,609
[Mn] + [S] = MnS(s)	(12)	26,948	26,936	26,824

**Table 7:** Melting points of Ce-containing inclusions.

Content	Ce_2_O_3_	CeS	Ce_2_S_3_	Ce_3_S_4_	Ce_2_O_2_S
Melting point °C	2177	2099	2149	2080 ± 30	1949

## Data Availability

The data presented in this study are available in this article.
